# Tetrandrine-Induced Autophagy in MDA-MB-231 Triple-Negative Breast Cancer Cell through the Inhibition of PI3K/AKT/mTOR Signaling

**DOI:** 10.1155/2019/7517431

**Published:** 2019-01-01

**Authors:** Yubo Guo, Xiaohua Pei

**Affiliations:** ^1^Beijing University of Chinese Medicine, Beijing 100029, China; ^2^The Fangshan Hospital, Beijing University of Chinese Medicine, Beijing 102488, China

## Abstract

The present study examined the effects of tetrandrine suppressing proliferation, targeting LC3, p62, and Beclin-1 autophagy genes by inhibiting PI3K/AKT/mTOR signaling in Triple-negative breast cancer (TNBC) MDA-MB-231 cell. Cell viability and apoptosis were evaluated by MTT and Annexin-V/PI double staining. Cytotoxicity was determined with LDH assay. Western Blot and Immunofluorescence were used to measure the protein levels of p62/SQSTM1, Beclin1, LC3-II/LC3-I, and PTEN/PI3K/AKT/mTOR signaling. Results showed that tetrandrine inhibited the MDA-MB-231 cell proliferation and induced the apoptosis. Tetrandrine at doses of 12.8, 16.1, and 25.7*μ*mol/L showed significant cytotoxicity on MDA-MB-231 cells (p<0.01). Tetrandrine induced MDA-MB-231 cell autophagy by decreasing p62/SQSTM1 expression, improving the expression of Beclin1 and LC3-II/LC3-I (p<0.01), inhibiting the PI3K/AKT /mTOR pathway by downregulating the expression of p-AKT ^ser473^/AKT, p-PI3K/PI3K p110*α*, and p-mTOR ^ser2448^/mTOR and upregulating PTEN expression. These findings revealed that tetrandrine could suppress proliferation and induce autophagy in MDA-MB-231 cell by inhibiting the PI3K/AKT/mTOR pathway and might be a promising anti-triple-negative breast cancer drug.

## 1. Introduction

Breast cancer is the most common women cancer and one of the leading causes of mortality in females [1]. Triple-negative breast cancer (TNBC), which accounts for approximately 10-20% of all breast cancers, has high proliferation, high histological grade, and poor prognosis [2]. Due to the lack of expression of ER, PR, and Her2/Neu, the current triple-negative breast cancer targeted therapy is ineffective and there are no established targeted agents for TNBC and basal-like BC. Although TNBC is sensitive to chemotherapy, compared with other subtypes, TNBC has a high recurrence rate with distant metastases, high metastasis rate, high recurrence rate, and poor overall survival [3].

Autophagy is a self-adaption and has a double-edged sword function in tumor metastasis. Autophagy has three types: macroautophagy, microautophagy, and chaperone-mediated autophagy. Autophagy in present study refers to macroautophagy [4]. Malignant tumor cells are usually apoptotic defects and autophagy is a form of cell death that is parallel to apoptosis. Autophagy may serve as an alternative mechanism for cell death when tumor cells have a defect in apoptosis or when apoptosis is inhibited [5]. The use of autophagy inducers can induce excessive autophagy of tumor cells and play synergistic effects on chemotherapeutic drugs beneficial to treatment [6]. Beclin1, LC3, and p62, the three major participants in autophagy, facilitate evaluation of autophagy levels of cells [7]. Studies have shown that LC3 and Beclin-1 are essential for cell proliferation, survival, migration, and invasion and may contribute to tumor growth and the development of highly invasive and metastatic TNBC cells. Targeted therapy of autophagy genes may be a potential therapeutic strategy in TNBC breast cancer [8].

Tetrandrine is a bisbenzylisoquinoline alkaloid extracted from the root of* Stephania tetrandra *S. Moore [9]. Although it has a wide range of pharmacological effects on lung cancer, nasopharyngeal carcinoma, laryngeal cancer, oral cancer, liver cancer [10–14], and so on, it has been demonstrated that tetrandrine has distinctive effect on breast cancer. For example, tetrandrine exhibited antitumor effects by inhibiting the growth of transplanted tumor and inducing the formation of apoptotic bodies of MCF-7 tumor-bearing nude mice [15]. Tetrandrine can reverse multidrug resistance of breast cancer cell MCF-7/Dox [16], enhance the cytotoxicity of Dox to MCF-7/Dox tumor cells, and significantly improve the reversal fold (RF) value [17, 18]. Besides, tetrandrine can enhance the sensitivity of tumor cells to high-energy radiation and DNA damage of anticancer drugs and exert a synergistic antitumor effect [19]. Tetrandrine also can play antitumor angiogenesis, antioxidative, and regulate the immune system function [20]. Although tetrandrine has a significant role in antibreast cancer, its mechanism on anti-triple-negative breast cancer (TNBC) remains unclear.

The PI3K/AKT/mTOR pathway has been shown to play significant roles in the development, progression, and metastatic of TNBC breast cancer [21, 22]. Mutations of the PTEN-PI3K-AKT axis occur in approximately 30% of breast cancers [23]. Besides, autophagy differs depending on the upstream regulatory signal pathway and is mainly dependent on mammalian rapamycin target protein mTOR signaling pathway. The typical AKT/protein kinase B mammalian rapamycin target (mTOR) signaling pathway is known to initiate vesicle bilayer formation in autophagy. AKT can activate autophagy-dependent classical mTOR pathway and inhibition of AKT/mTOR pathway can induce autophagy and help block breast cancer progression. The PI3K, which is particularly relevant to cancer, converts PIP2 to PIP3. PIP3 binds to AKT and PDK1, causing PDK1 to phosphorylate Ser308 and Ser473 of the AKT protein, resulting in activation of AKT. AKT phosphorylates TSC1/2 and activates the mTOR complex (mTORC1) to activate the translation of proteins and enhances the growth of cancer cells. PTEN is a PIP3-phosphatase that functions in contrast to PI3K, which converts PIP3 to PIP2 by dephosphorylating. Around 40% of BC shows loss of expression of PTEN, especially in hormone-receptor- (HR-) negative breast cancer [24]. It is reported that tetrandrine might lead to autophagic induction through PKC-*α* inactivation [25]. However, it is unclear whether tetrandrine can induce autophagy in triple-negative breast cancer cells and whether its mechanism inhibits PI3K/AKT/mTOR pathway.

In light of these findings, we investigate whether tetrandrine could suppress proliferation in human triple-negative breast cancer MDA-MB-231 cell targeting autophagy and its potential association with the PTEN/PI3K/AKT/mTOR signaling pathway.

## 2. Materials and Methods

### 2.1. Chemicals and Reagents

Tetrandrine was purchased from National Institutes for Food and Drug Control. RPIM was purchased from Gibco Life Technologies, Grand Island, NY, USA (lot. No: 1894129). Fetal bovine serum (FBS) was purchased from ExCell Bio Inc., Australia (lot. No: 11G047). Penicillin Streptomycin (100 Units/mL Penicillin and 100*μ*g/mL Streptomycin) was purchased from Gibco Life Technologies, Grand Island, NY, USA (lot. No: 1857814). ly294002 (a specific inhibitor of PI3K) was purchased from Selleck.cn, Houston, Texas, USA (lot. No: S110503), which served as a positive control for this experiment. LDH assay kit was purchased from Nanjing Jiancheng Bioengineering Institute (Cat. No: A020-2). BCA protein quantitation assay and whole cell lysis assay were purchased from Nanjing Keygen Biotech. Co., Ltd. (Cat. No: KGPBCA, Cat. No: KGP103). Rabbit anti-LC3 Polyclonal Antibody (Cat. No: 12135-1-AP), Rabbit anti-p62/SQSTM1 Polyclonal Antibody (Cat. No: 18420-1-AP), Rabbit anti-Beclin1 Polyclonal Antibody (Cat. No: 11306-1-AP), Mouse anti-PTEN Monoclonal Antibody (Cat. No: 60300-1-Ig), Mouse anti-PI3K p85 (alpha) Monoclonal Antibody (Cat. No: 60225-1-Ig), Rabbit anti-PI3K p110 (alpha) Polyclonal Antibody (Cat. No: 21890-1-AP), Mouse anti-AKT Monoclonal Antibody (60203-2-Ig), and Mouse anti-beta Actin Monoclonal antibody (Cat. No: 66009-1-Ig) were purchased from Proteintech Group, Inc. (Chicago, USA). Rabbit anti-Phospho-PI3 Kinase p85 (Tyr458)/p55(Tyr199) Polyclonal Antibody and Rabbit anti-Phospho-Akt(Ser473) (D9E)XP® Monoclonal Antibody were purchased from Cell Signaling Technology, Inc. (Boston, MA, USA), and Mouse Anti-mTOR Monoclonal Antibody[53E11] ab87540 and Rabbit Anti-mTOR(phosphor S2448) Monoclonal antibody [EPR426(2)](ab109268) were purchased from Abcam Biocompany (Cambridge, MA, USA). FITC conjugated Annexin-V apoptosis detection kit instructions (Becton, Dickinson and Company, Franklin Lake, New Jersey, USA, lot. No: 7040932). All other reagents were obtained from Sigma-Adrich Co. (St. Louis, MO, USA). The standard of tetrandrine was dissolved in DMSO (concentration <0.1%) before adding to the RPMI-1640 culture medium making the final concentration 64.2 *μ*mol/L, protected from light and stored in a refrigerator at -20°C.

### 2.2. Cell Cultures

Human breast cancer cell MDA-MB-231 was purchased from Cell Resource Center, Shanghai Institutes for Biological Sciences, Chinese Academy of Sciences, and cultured with PRIM 1640 (Gibco Life Technologies, Grand Island, NY, USA), 10% fetal bovine serum (FBS, ExCell Bio Inc., Australia) and 1% Penicillin Streptomycin at 37°C with 5% CO^2^ in a humidified cell incubator under 95%/5% (v/v) mixture of air and CO^2^.

### 2.3. Assessment of Cell Viability by MTT Method

The cell viability of human breast cancer cell MDA-MB-231 treated with tetrandrine was investigated using MTT assay. MDA-MB-231 cells (5×10^4^ cells/ml) were seeded in 96-well plates and cultured with normal medium to adherent. Cells were treated with tetrandrine at doses of 0, 0.8, 1.6, 3.2, 6.4, 12.8, 16.1, 25.7, 32.1, 51.4, and 64.2*μ*mol/L for 24h, 48h, and 72h. A volume of 20*μ*l of MTT 5mg/ml was added to each well and incubated for another 4h at 37°C. The medium was then aspirated carefully without disturbing the blue formazan crystals. DMSO was added (150*μ*l/well) shaking for 15min. The optical density (OD) value was measured at 490 nm on a multifunctional microplate reader (FLUO star Omega, BMG Labtech, Germany). The results were expressed as a percentage of the absorbance present in treated cells compared with control cells. Cell proliferation inhibition rate formula: [1-(drug group OD value/Control group OD value)] × 100%. The IC50 of 72h was calculated using GraphPad Prism 5.01 software.

### 2.4. Apoptosis Assessment by Flow Cytometry

Cells were seeded in 6-well plates at a density of 1×10^6^/well and treated with different concentrations of tetrandrine for 72h. Cells were harvested without EDTA-trypsin digestion solutions (Solarbio science & technology Co., Ltd., Beijing, lot. No: 20171024), washed twice with PBS, and resuspended in 200*μ*l Binding Buffer, Pipette 100 *μ*l of the cell suspension into Eppendorf tube 1.5ml). The cell suspension was mixed with 5*μ*l of Annexin-V-fluorescein isothiocyanate (FITC) and 5*μ*l Propidium Iodide (PI) according to the FITC conjugated Annexin-V apoptosis detection kit instructions (Becton, Dickinson and Company, Franklin Lake, New Jersey, USA, lot. No: 7040932), incubated for 15 min in the dark at room temperature. Then 150*μ*l binding buffer was added to each tube and they were analyzed with a flow cytometer within 1 hour.

### 2.5. The Cytotoxicity Assessment by LDH Assay

LDH assay was used to test the cell toxicity of tetrandrine on human breast cancer MDA-MB-231 cell. Cells were seeded in 6-well plates at a density of 1×10^6^/well and treated with different concentrations of tetrandrine for 48h. The cell supernatant was collected and 0.2*μ*mol/ml standard solutions of pyruvate were both added 20*μ*l to a 96-well plate. Set up blank group and Control group. According to LDH kit instructions, mix with matrix buffer 25*μ*l and the cell supernatant sample group was added in coenzyme 1 application solution 5*μ*l. Mix well and incubate at 37°C for 15min. Add 2,4-dinitrophenylhydrazine 25*μ*l to the 96-well plate. Mix well and incubate at 37°C for 15min. Then it was the 0.4 mol/l NaOH solution 250*μ*l. Mix well and stand at room temperature 5min. The optical density (OD) value was measured at 450 nm on a multifunctional microplate reader (FLUOstar Omega, BMG Labtech, Germany).

### 2.6. Immunofluorescence Analysis

The logarithmic growth phase of MDA-MB-231 cells was made into cell suspension, dropping 500*μ*l (1×10^6^/well) into a glass bottom cell culture dish with 20mm diameter and incubating it 4-5h for adherent cells. It was treated with different concentrations of tetrandrine for 48h and fixed with 4% precooled paraformaldehyde for 20 minutes, Permeate with 0.2% Triton X-100 for 10 minutes. 10% Goat serum blocked them for 30 minutes. Incubate primary antibody and put into the wet box overnight at 4°C. Wash three times with PBS. Fluorescent secondary antibody was incubated at room temperature for 30min (away from light). Wash three times with PBS. DAPI (Solarbio, lot. No: 20170815) stained nuclei. Use the Laser confocal scanning microscope to take pictures and observe the protein expression and their analysis was with Image Pro Plus software 6.0.

### 2.7. Western Blot Analysis

The total protein of cells was extracted by protein extraction kit and quantified by BCA method conducted according to the procedure as previously described [26] with some modifications. Briefly, 20 *μ*g of protein from the MDA-MB-231 cell was on 10% polyacrylamide gel by SDS-PAGE electrophoresis (concentration gel for 30min, separation gel for 1.5h) followed by transferring onto PVDF membrane (Immobilon®-P Transfer Membranes, 0.45*μ*m, lot. No: K5PA9282A). Then the membrane was subsequently incubated with appropriate primary antibody (1:500) for overnight at 4°C, and the corresponding HRP labeled secondary antibodies at room temperature for 1h. Add ECL chemiluminescence solution (Proteintech, lot. No: B500023) on the membrane, with a gel imager (Azure c500, Azure Bio systems, USA) exposure. Grayscale values were analyzed with ImageJ and internal controls were used with *β*-actin.

### 2.8. Statistical Analysis

All results were expressed as mean ± standard deviation (SD). One-way ANOVA was performed between multiple groups using SPSS software (Version 20.0) when homogeneity of variance and normality were met. Otherwise, Dunnett's T3 and nonparametric tests were conducted between multiple groups, respectively. p<0.05 was considered statistical difference and p<0.01 was considered statistical significant difference.

## 3. Results

### 3.1. Effects of Tetrandrine on MDA-MB-231 Cell Viability

**Figure 1:**
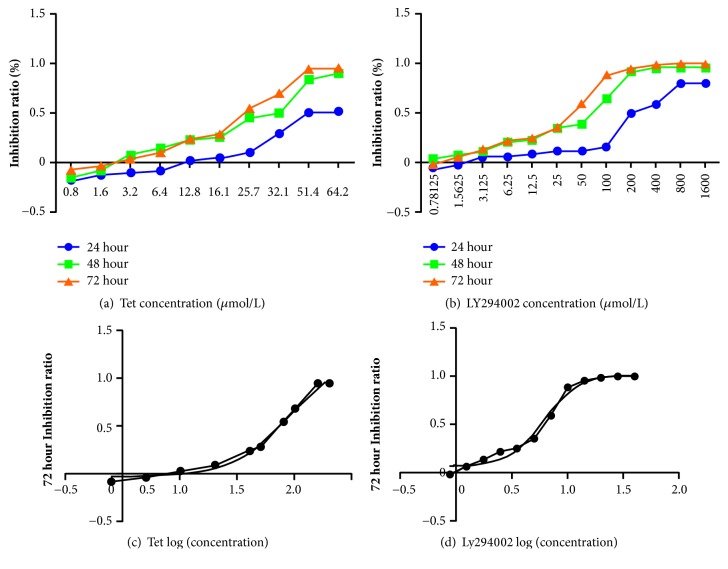
The inhibition rate and IC50 of tetrandrine and LY294002 on MDA-MB-231 cells.

MTT method was used to detect the inhibition rate of tetrandrine in human breast cancer MDA-MB-231 cell proliferation. MDA-MB-231 cell was respectively treated with tetrandrine and LY294002. Results showed that tetrandrine at doses of 6.4, 12.8, 16.1, 25.7, 32.1, 51.4, 64.2*μ*mol/L inhibited the MDA-MB-231 cell proliferation, as shown in Figure 1(a). The IC50 at 72 hours of tetrandrine on MDA-MB-231 breast cancer cells proliferation was 28.06*μ*mol/L, with 95% confidence interval (17.81-44.20) (Figure 1(c)). LY294002 at doses of 3.125, 6.25, 12.5, 25, 50, 100, 200, 400, 800, and 1600*μ*mol/L inhibited the MDA-MB-231 cell proliferation, as shown in Figure 1(b). The IC50 at 72 hours of LY294002 functioned on MDA-MB-231 cell was 38.32, with 95% confidence interval (27.17-54.06) (Figure 1(d)).

Figures 1(a)–1(d) showed inhibition rate of cell proliferation and IC50 of tetrandrine and LY294002. MTT method was used to measure the cell proliferation inhibition rate. Different doses of tetrandrine (0, 0.8, 1.6, 3.2, 6.4, 12.8, 16.1, 25.7, 32.1, 51.4, 64.2*μ*mol/L) and LY294002 (0, 0.78125, 1.5625, 3.125, 6.25, 12.5, 25, 50, 100, 200, 400, 800, 1600*μ*mol/L) were given to MDA-MB-231 cell for 24, 48, and 72 hours.

### 3.2. Effects of Tetrandrine on MDA-MB-231 Cell Apoptosis

**Figure 2:**
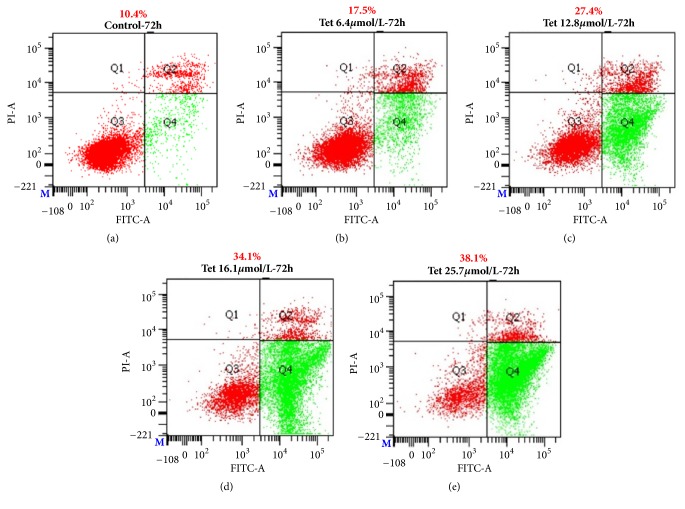
Comparison of apoptosis rate in different concentrations of tetrandrine group in human breast cancer MDA-MB-231 cells with Annexin-V and PI double staining.

Cells (2×10^5^ cells/well) were incubated with or without tetrandrine at doses of 0, 6.4, 12.8, 16.1, and 25.7*μ*mol/L for 72h and then were harvested for staining with Annexin-V and PI double staining to measure the percentage of apoptotic cells by flow cytometry. MDA-MB-231 cell was treated with or without tetrandrine for 72h. Tetrandrine at doses of 0, 6.4, 12.8, 16.1, and 25.7*μ*mol/L induced MDA-MB-231 cell apoptosis rate of 10.4%, 17.5%, 27.4%, 34.1%, and 38.1%, respectively. Compared with the Control group, the tetrandrine group of the normal quadrant were significantly decreased and the early apoptotic quadrant were significantly increased (p<0.01), as shown in Figure 2 and Table 1.

Figures 2(a)–2(e) and Table 1 showed the cell apoptosis rate in different concentrations of tetrandrine groups. Annexin-V and PI double staining was used to measure the cell apoptosis. Different doses of tetrandrine (0, 6.4, 12.8, 16.1, 25.7*μ*mol/L) were given to MDA-MB-231 cell for 72 hours (Table 1*∗*compared with the Control group; p<0.05 was considered statistical difference and p<0.01 was considered significant difference).

### 3.3. Effects of Tetrandrine on MDA-MB-231 Cytotoxicity

**Figure 3:**
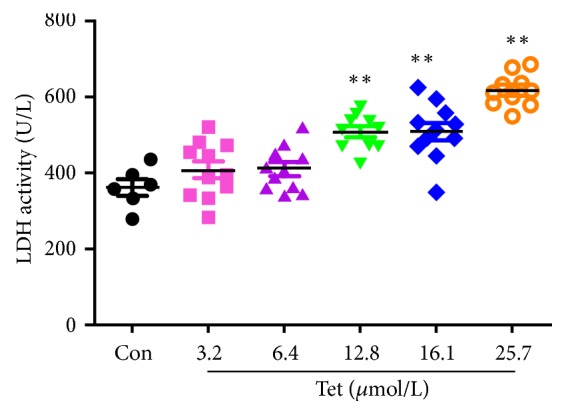
Comparison of the cytotoxicity of different concentrations of tetrandrine in MDA-MB-231 cell.

LDH (lactate dehydrogenase) is a stable protein that exists in the cytoplasm of normal cells. Once the cell membrane is damaged, LDH is released to the outside of the cell to increase the LDH activity in the extracellular fluid. Compared with Control group, tetrandrine at doses of 12.8, 16.1, and 25.7*μ*mol/L showed obvious cytotoxicity to human breast cancer MDA-MB-231 cell (p<0.01, Figure 3).

Figure 3 showed the cytotoxicity in different concentrations of tetrandrine groups. LDH assay was used to measure the cytotoxicity. Different doses of tetrandrine (0, 3.2, 6.4, 12.8, 16.1, and 25.7*μ*mol/L) were given to MDA-MB-231 cell for 48 hours (Figure 3*∗*compared with the Control group; p<0.05 was considered statistical difference and p<0.01 was considered significant difference).

### 3.4. Effects of Tetrandrine on Beclin1, LC3, and p62/SQSTM1 Expression

**Figure 4:**
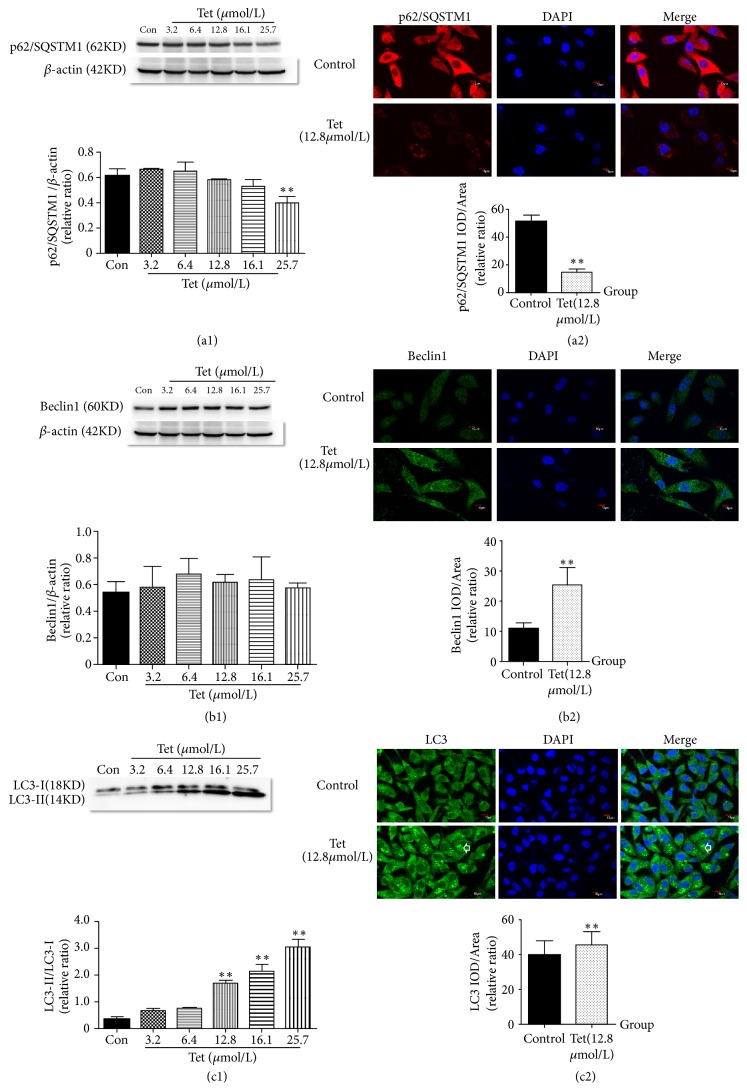
Comparison of p62/SQSTM1, Beclin1, and LC3 in MDA-MB-231 cells intervened with different concentrations of tetrandrine.

Previous studies [25] have shown that tetrandrine can induce autophagy in tumor cells. In order to observe whether tetrandrine could induce autophagy in triple-negative breast cancer cells and select appropriate concentration of tetrandrine, Beclin1, LC3, and p62/SQSTM1, the three major proteins involved in the autophagy process, were examined by Western Blot analysis and also confirmed by immunofluorescence staining assay. Results of Western Blot showed that tetrandrine at dose of 25.7*μ*mol/L decreased p62/SQSTM1 (p<0.01) and at doses of 12.8, 16.1, and 25.7*μ*mol/L improved LC3-II/LC3-I expression (p<0.01) as shown in Figures 4(a1)–4(c1). Immunofluorescence staining had shown that p62/SQSTM1 and the autophagy flow of LC3 were distributed in the cytoplasm, while Beclin1 was expressed in the cytoplasm and nucleus. Tetrandrine (12.8*μ*mol/L) improved the activation of autophagy flow of LC3 and Beclin1 expression and inhibited the expression of p62/SQSTM1 in MDA-MB-231 cell. It was found that tetrandrine could induce autophagy in triple-negative breast cancer cells. With the increase of concentration, Tet induced the autophagy of human triple-negative breast cancer cells MDA-MB-231.

Figures 4(a)–4(c) showed Beclin1, LC3, and p62/SQSTM1 protein expression of MDA-MB-231 with different concentrations of tetrandrine intervened. Immunofluorescence staining was used to measure the protein expression of Beclin1, LC3, and p62/SQSTM1, which were observed with the laser confocal scanning microscope (original magnification, ×600) (Figures 4(a2)–4(c2)) and their analysis was with Image Pro Plus software 6.0. White arrow in Figure 4(c2) represents autophagy flow. Western Blot was used to measure the protein levels of Beclin1, LC3, and p62/SQSTM1 expression and their analysis was with ImageJ. Different concentrations of tetrandrine (0, 3.2, 6.4, 12.8, 16.1, and 25.7*μ*mol/L) were given to MDA-MB-231 cell for 48 hours (Figures 4(a1)–4(c1)*∗*compared with the Control group; p<0.05 was considered statistical difference and p<0.01 was considered significant difference).

### 3.5. Effects of Different Concentrations of Tetrandrine on PI3K/AKT/mTOR Pathway in MDA-MB-231 Cells

**Figure 5:**
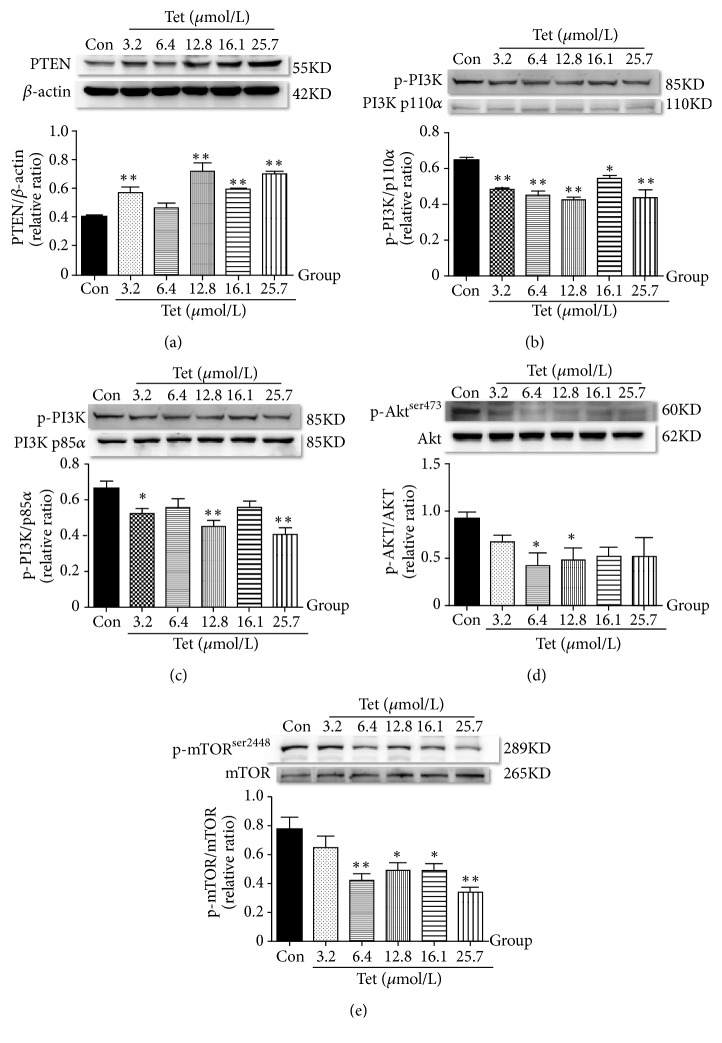
Comparison of the protein levels of PI3K/AKT/mTOR pathway in MDA-MB-231 cells intervened with different concentrations of tetrandrine.

Increased activity of the PI3K/AKT pathway is often associated with multiple cancers. Autophagy relies mainly on the mammalian target of rapamycin (mTOR) pathway. We investigated the effect of tetrandrine on the PI3K/AKT/mTOR signaling pathway in vitro by incubating MDA-MB-231 cells with different concentrations of tetrandrine (0, 3.2, 6.4, 12.8, 16.1, and 25.7*μ*mol/L) for 48h. Western Blot analysis, as shown in Figures 5(a)–5(e), indicated that tetrandrine could reduce the expression of p-akt_ _^ser473^ /akt, p-PI3K/PI3K p110*α*, p-PI3K/PI3K p85*α*, p-mTOR_ _^ser2448^ /mTOR and improved the expression of PTEN (p<0.05,0.01) compared with Control group, inhibiting the PI3K-AKT-mTOR pathway.

Figure 5 showed the protein levels of p-akt_ _^ser473^ , akt, p-PI3K, PI3K p110*α*, p-PI3K, PI3K p85*α*, p-mTOR_ _^ser2448^ , mTOR in different concentrations of tetrandrine groups. The Western Blot method was used to measure the protein expression levels of the PTEN/PI3K/AKT/mTOR pathway and their analysis was with ImageJ. Different concentrations of tetrandrine (0, 3.2, 6.4, 12.8, 16.1, and 25.7*μ*mol/L) were given to MDA-MB-231 cell for 48 hours (Figures 5(a)–5(e)*∗*compared with the Control group; p<0.05 was considered statistical difference and p<0.01 was considered significant difference).

### 3.6. Effects of Tetrandrine (12.8*μ*mol/L) and ly294002 (50*μ*mol/L) Inhibitor of PI3K/AKT Pathway on MDA-MB-231 Cells

**Figure 6:**
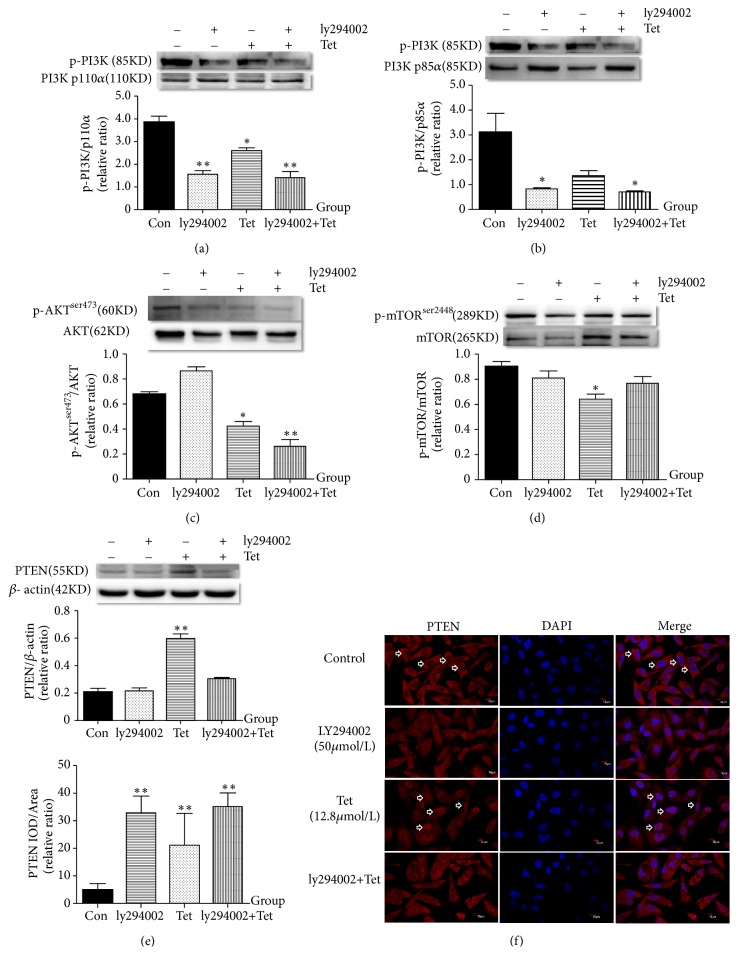
Comparison of the protein levels of PTEN/PI3K/AKT/mTOR pathway in different groups.

To further examine whether tetrandrine-induced autophagy involves the PI3K/AKT/mTOR signaling pathway, we pretreated MDA-MB-231 cells with tetrandrine (12.8*μ*mol/L) and ly294002 (50*μ*mol/L) inhibitor of PI3K/AKT signaling pathway and then the levels of AKT, p-AKT _ _^ser473^ , p110*α*, p-PI3K, mTOR, and p-mTOR_ _^ser2448^  were examined. As shown in Figures 6(a)–6(e), results indicated that tetrandrine (12.8*μ*mol/L) could inhibit the PI3K/AKT/mTOR signaling pathway by improving expression of PTEN and reducing expression of p-AKT _ _^ser473^ /AKT, p-PI3K/p110*α*, p-mTOR_ _^ser2448^ /mTOR (p<0.05,0.01). Furthermore, the immunofluorescence staining results, as shown in Figure 6(f), indicated that, in the blank group, PTEN was only expressed in the cytoplasm of MDA-MB-231 cell. After intervention with tetrandrine (12.8*μ*mol/L), PTEN was expressed both in the nucleus and cytoplasm. PTEN protein level in tetrandrine group was significantly higher than the Control group (p<0.01), while lower than the LY294002 group (p<0.01).

Figures 6(a)–6(f) showed the protein levels of p-akt_ _^ser473^ , akt, p-PI3K, PI3K p110*α*, p-PI3K, PI3K p85*α*, p-mTOR_ _^ser2448^ , mTOR in different concentrations of tetrandrine groups. The Western Blot method was used to measure the protein expression levels of the PTEN/PI3K/AKT/mTOR signaling and their analysis was with ImageJ. Immunofluorescent staining, observed with the laser confocal scanning microscope (original magnification, ×600) (f), was used to measure the PTEN expression and the analysis was with Image Pro Plus software 6.0. The PTEN expression in MDA-MB-231 cell nucleus was indicated with the white arrows. Tetrandrine (12.8*μ*mol/L) and ly294002 (50*μ*mol/L) were given (Figure 6, *∗*compared with the Control group; p<0.05 was considered statistical difference and p<0.01 was considered significant difference).

## 4. Discussion

This study revealed that tetrandrine could inhibit the proliferation of triple-negative breast cancer MDA-MB-231 cells, induce early apoptosis, and have certain toxicity to MDA-MB-231 cell in a concentration and time dependent manner. The mechanism is probably due to the fact that tetrandrine could induce MDA-MB-231 cell autophagy by reducing the expression of p62 and increasing the expression of Beclin1, LC3-II/LC3-I. The triple-negative breast cancer MDA-MB-231 cells autophagy induced by tetrandrine was by inhibiting the PI3K/AKT/mTOR signaling via upregulating PTEN expression and downregulating p-akt_ _^ser473^ /akt, p-PI3K/PI3K p110*α*, p-mTOR_ _^ser2448^ /mTOR. LY294002 inhibited PI3K/AKT signaling pathway by reducing p-PI3K/p85*α* and p-PI3K/p110*α*, to inhibit breast cancer cell proliferation and induce apoptosis.

Our previous study found that tetrandrine had a significant inhibitory effect on MCF-7 xenograft tumor in nude mice. The nude mice were subcutaneously inoculated with MCF-7 breast cancer cells in the right axilla of the nude mice, and the nude mice model of breast cancer transplanted tumor was inoculated for 7 days. Tetrandrine treatment was given for 15 days. It was found that tetrandrine had obvious antitumor growth effect and induced breast cancer cell apoptotic bodies in vivo [15]. Furthermore, in vitro experiments, it is reported that tetrandrine combined with cisplatin was applied to breast cancer cell MDA-MB-231 for 24 hours, the surface structure of the cell membrane was destroyed, and pores were formed. After 48 hours, the cell membrane was severely damaged. The cell cycle ratio increased to 51.7% in S phase, which improved the anticancer drugs of cancer cells sensitivity [27]. Consistent with the results of the previous study, this study found that tetrandrine could inhibit the proliferation of triple-negative breast cancer cells MDA-MB-231, inducing early apoptosis after 72h (p <0.01).

Autophagy is a double-edged sword for tumor therapy. One is that autophagy protects tumor cells and reduces the adverse effects of the surrounding environment. The other is to upregulate the autophagy activity of tumor cells and initiate programmed cell death. Studies have shown that knocking down the autophagy genes LC3 and Beclin1 could lead to a significant decrease in autophagy and inhibit the proliferation, migration, and invasion of triple-negative breast cancer MDA-MB-231 cells [8]. Tetrandrine, which can reverse the multidrug resistance of tumor cells, inhibiting tumor angiogenesis and playing an antitumor effect, is a potent cell autophagy agonist [28]. In this study, we found that tetrandrine could inhibit the proliferation of MDA-MB-231 cells and induce autophagy and apoptosis by decreasing p62/SQSTM1 expression and increasing Beclin1 and LC3 expression, thereby reversing the protective effect of autophagy on TNBC cells. The results of our study showed that tetrandrine could activate autophagy and induce autophagic death in TNBC cells.

To further reveal the activating autophagy mechanism of tetrandrine, the PI3K/AKT/mTOR pathway was considered. It was reported that TNBC was subclassified into at least six tumor molecular subtypes including a mesenchymal-like subset that was highly sensitive to PI3K/mTOR inhibitors in vitro and in vivo [24]. Our study showed that LY294002, as the PI3K inhibitor which served as a positive control for this experiment, could reduce p-PI3K/p85*α* and p-PI3K/p110*α*, increase the expression of PTEN, and block the PI3K/AKT pathway to inhibit the triple-negative breast cancer MDA-MB-231 cell proliferation, which suggested that the inhibitor of PI3K/AKT pathway played an important role in the development of breast cancer. Moreover, our research found that tetrandrine was also an inhibitor of PI3K/AKT/mTOR signaling pathway and could significantly inhibit the proliferation and induce apoptosis in triple-negative breast cancer MDA-MB-231 cells.

The phosphoinositol-3-kinase family is divided into four different classes: Class I, Class II, Class III, and Class IV. Among them, the most widely studied is Class I PI3K. Class IA PI3K is composed of a heterodimer between a p110 catalytic subunit and a p85 regulatory subunit. The ways in which p85 subunits contribute to cancer and the effective means to pharmacologically inhibit these mechanisms are still unclear [29]. The gene encoding for PI3K catalytic subunit p110*α* is mutated in 20-40% of breast cancer [30]. In our study, it was found that tetrandrine could inhibit p-PI3K/PI3K p110*α*, which provided a novel drug for the study of PIC3A gene mutation of TNBC. Therefore, tetrandrine inhibited the triple-negative breast cancer MDA-MB-231 cell proliferation and induced autophagy likely by the inhibition of PI3K/AKT/mTOR signaling pathway.

PTEN is a tumor suppressor gene that inhibits cell proliferation by inhibiting the phosphoinositide 3-kinase (PI3K) signaling pathway [31]. PTEN deletion was significantly associated with estrogen receptor negative (ER-), especially in triple-negative breast cancer [32]. Studies had shown that degrading PTEN through lysosome-mediated activation of PI3K/AKT/GSK3*β*/SNAI1 signaling pathway could promote the metastasis and progression of EMT and breast cancer tumors, which showed that the loss of PTEN contributed to the development of breast cancer [33]. In our study, tetrandrine could significantly increase PTEN content compared with the Control group (p<0.01) by expressing both nucleus and cytoplasm, inhibiting the progression of TNBC. These data suggested that tetrandrine might be a PTEN enhancer, which provide clinical targeted drugs for Triple-negative breast cancer.

However, some limitations should be noted in the current study. It is reported that hyperactivation of the phosphatidylinositol 3-kinase/AKT/mammalian target of rapamycin (PI3K/AKT/mTOR) pathway is implicated in the tumor genesis of ER+ breast cancer and in resistance to endocrine therapy [34]. Our future endeavors still need to further explore the relationship between tetrandrine with ER+ breast cancer and endocrine therapy resistance. Besides, tetrandrine as the inhibitor of PI3K/AKT/mTOR also needs to be further exploited through rational combinations with immunotherapies and targeted therapies to improve Triple-negative breast cancer clinical outcomes.

## 5. Conclusions

In conclusion, we have proposed a novel mechanism of tetrandrine inducing autophagy on the triple-negative breast cancer MDA-MB-231 cells. High expression of p62 and low expression of Beclin1 and LC3-II/LC3-I in the human breast cancer MDA-MB-231 cells lead to autophagy and apoptosis defects which accelerate breast cancer progression. Intervention on MDA-MB-231 cell with tetrandrine inhibits the proliferation and induces autophagy through inhibiting the PI3K/AKT/mTOR signaling via upregulating PTEN expression and downregulating p-akt_ _^ser473^ /akt, p-PI3K/PI3K p110*α*, p-mTOR_ _^ser2448^ /mTOR, suggesting tetrandrine may serve as a promising active antitumor drug, by a direct regulation of the PI3K/AKT/mTOR pathway in the triple-negative breast cancer MDA-MB-231 cell. The present paper also warrants further study of tetrandrine in the treatment of triple-negative breast cancer with autophagocytosis and targeted therapy of chemotherapeutic drugs.

## Figures and Tables

**Table 1:** Comparison of apoptosis rate in different concentrations of tetrandrine group in MDA-MB-231 cells (Mean±SD) (n=3).

**Group**	**Normal cell(**%**)**	**Early apoptosis(**%**)**	**Late apoptosis(**%**)**	**Necrosis(**%**)**
**Control**	70.65±1.63	5.00±0.71	5.35±0.78	0.25±0.07
**Tet (6.4**μ**mol/L)**	49.85±1.77_ _^*∗∗*^	10.45±0.21_ _^*∗∗*^	6.80±0.57	0.55±0.07_ _^*∗*^
**Tet (12.8**μ**mol/L)**	25.05±1.91_ _^*∗∗*^	20.75±0.92_ _^*∗∗*^	5.70±0.42	0.25±0.07
**Tet (16.1**μ**mol/L)**	13.10±1.56_ _^*∗∗*^	29.95±0.49_ _^*∗∗*^	3.45±0.49	0.35±0.07
**Tet (25.7**μ**mol/L)**	8.70±1.13_ _^*∗∗*^	33.90±0.42_ _^*∗∗*^	3.40±0.71	0.25±0.07

## Data Availability

Authors make data supporting the conclusions of the study available to all interested researchers upon request through the authors themselves. Xiaohua Pei should be contacted to request the data and the email address is pxh_127@163.com.
